# Detection of virus-specific polymeric immunoglobulin A in acute hepatitis A, C, E virus serum samples using novel chimeric secretory component

**DOI:** 10.1186/s13104-018-3799-2

**Published:** 2018-10-01

**Authors:** Khayriyyah Mohd Hanafiah, Mary L. Garcia, Nadine C. Barnes, David A. Anderson

**Affiliations:** 1 Life Sciences, Macfarlane Burnet Institute, 85 Commercial Rd, Melbourne, VIC 3004 Australia; 2 Department of Immunology, Monash University, 86 Commercial Road, Melbourne, VIC 3004 Australia; 3 School of Biological Sciences, Universiti Sains Malaysia, Gelugor, Penang 11800 Malaysia; 4 Department of Microbiology and Immunology, University of Melbourne, 792 Elizabeth Street, Melbourne, VIC 3000 Australia

**Keywords:** Polymeric immunoglobulin A, Polymeric immunoglobulin receptor, Secretory component, Biomarkers, Serodiagnostics, Hepatitis A virus, Hepatitis E virus, Hepatitis C virus

## Abstract

**Objective:**

To conduct a proof-of-concept study on preferential binding of polymeric IgA (pIgA) using a novel recombinant rabbit/human chimeric secretory component (cSC) and preliminary assessment of the diagnostic potential of virus-specific pIgA in discriminating acute hepatitis A, E, and C (HAV, HEV, HCV) patients and uninfected controls using an indirect enzyme-linked immunoassay.

**Results:**

cSC binds > 0.06 μg/ml of purified human and mouse pIgA with negligible cross-reactivity against IgM and IgA. Virus-specific pIgA was significantly higher in serum of acute HAV (n = 6) and HEV (n = 12) patients than uninfected samples (HEV: p < 0.001; HAV: p = 0.001), and had low correlation with virus-specific IgM (HEV r: − 0.25, 95% CI − 0.88 to 0.71, p = 0.636; HAV r: 0.05, 95% CI − 0.54 to 0.60, p: 0.885). Anti-HCV pIgA peaked early in HCV seroconversion panels (n = 14), and was undetectable after 4 weeks post-primary bleed, even in ongoing infections, while serum anti-HCV IgA, IgG and IgM persisted. Patients with early acute HCV infection had significantly higher levels of anti-HCV pIgA compared to those with chronic infections (p < 0.01). The use of novel cSC demonstrates the presence of virus-specific pIgA in sera of patients with acute HAV, HEV, and HCV infection, and posits its potential utility as a diagnostic biomarker that warrants further validation on larger sample populations.

## Introduction

Viral hepatitis contributes significant global disease burden [1]. Hepatitis A and hepatitis E viruses (HAV, HEV) are enterically transmitted but replicate in and cause acute inflammation of the liver [2, 3], while parenterally transmitted hepatitis C virus (HCV) causes chronic hepatitis in 75–85% of infected individuals [4]. These infections begin and/or persist in mucosal tissues where polymeric immunoglobulin (Ig) A (pIgA) is the predominant antibody produced [5–7]. PIgA is transported by the polymeric immunoglobulin receptor (pIgR) to the epithelial surface where the pIgA-bound secretory component (SC) of pIgR is cleaved, releasing secretory IgA (SIgA) [5, 8–10]. Anti-HAV and anti-HEV IgA have been reported in the acute phase of disease [2, 3], but the proportion of pIgA is unknown. Anti-HCV IgM cannot discriminate chronic from acute HCV infections; and IgG cannot discriminate current from past HCV infections, and less is known on the role of anti-HCV IgA. With HEV being recognised as an emerging disease in industrialized countries [11, 12], chronic HCV causing of mortality from liver cancer and cirrhosis worldwide [1] and HAV a major source of food-borne outbreaks [13], there is interest in improved biomarkers to diagnose these infections.

While entirely polymeric in most animals, only 1–15% of human serum IgA is pIgA, the rest is monomeric [14–18]. Previous studies of antigen-specific pIgA in human disease relied on gel filtration to separate pIgA [19, 20]—cumbersome for translational studies of immune responses. Consequently, the role of pIgA as a diagnostic biomarker remains underexplored. In this study, a recombinant chimeric SC (cSC) was expressed based on described methods [21–23], and a novel enzyme-linked immunoassay (ELISA) was designed to enable preferential binding of low amounts of pIgA present in patient sera (≈ 0.2 mg/ml dIgA versus ≈ 1 mg/ml IgM). Using the cSC-based ELISA, pIgA responses in HAV, HCV, HEV infections were examined as proof-of-concept for serodiagnostic application in viral hepatitis.

## Main text

### Materials and methods

#### Sample population

ELISA-confirmed anti-HEV IgM+ acutely infected (n = 6) and uninfected sera (n = 8) were from a Nepalese prison study (Dr IL Shrestha, Siddhi Polyclinic), and anti-HAV IgM+ acutely infected (n = 12) and healthy sera (n = 4) were commercially sourced (BBI Diagnostics, SeraCare; West Bridgewater, MA) and from Alfred Hospital, respectively. HCV ribonucleic nucleic acid (RNA)-confirmed early incident seroconversion panels (n = 14), patients chronically infected/RNA+ > 6 months (n = 5), patients who spontaneously cleared HCV after 6 months/late clearers (n = 5) and uninfected/RNA− controls (n = 5) were from the HITS-i cohort study [24] (Professor Andrew Lloyd) and commercially sourced (n = 5) (BBI diagnostics). Samples were de-identified and analyzed anonymously, with approval from the Alfred Ethics Committee (581/14).

#### Cloning and expression

Soluble cSC, 6XHistidine-tagged cSC (cSC-His) and human CD4 cytoplasmic domain (D)-containing cSC (cSC-CD4), human SC (hSC-CD4) and rabbit SC (rSC-CD4) were expressed using modified published methods [22]. The hSC and rSC sequences were obtained from Genebank NM_002644.3 and X00412.1, respectively. Chimera of rSC-D1/hSC-D2-D5 were generated by splice overlap extension polymerase chain reaction with primers that introduced silent mutations in D1/D2 overlaps, followed by rSC/hSC-D1 exchange using *Eco*RI and *Sac*I restriction digestion, and cloning in eukaryotic expression vector pCDNA3.1 Zeo (Invitrogen; San Diego, CA). Constructs were confirmed by DNA sequencing. Human embryonic kidney 293T (HEK293T) cells [25] were grown in Dulbecco’s Modified Eagle Medium (DMEM)-GlutaMAX, 2.5% foetal calf serum (FCS), 100 U/ml Penicillin and 100 μg/ml Streptomycin (Invitrogen; San Diego, CA). HEK293T cells were transfected with plasmid encoding rSC-D1/hSC-D2-D5 using Lipofectamine 2000 (Invitrogen; San Diego, CA) based on manufacturer’s protocol, plus 25 ml DMEM-GlutaMAX + 10% FCS + 1% Penicillin/Streptomycin. The cSC-containing supernatants were harvested 48–72 h post-transfection and centrifuged to remove cells.

#### Gel and western blot

Samples mixed with 2xLaemmli reducing loading buffer, boiled, and electrophorosed in 4–15% Mini-Protean TGX precast polyacrylamide gel (BioRad; Hercules, California) for 40 min at 150 V were dry-blotted to nitrocellulose membranes using iBlot^®^ (Life Technologies; Carlsbad, California). Membranes were incubated rolling in 5% skim milk in phosphate buffered saline (PBS)-0.05% Tween-20 (Amresco; Solon, OH) for 1 h at RT, then in mouse monoclonal anti-human SC (1 μg/ml) (Abcam; Abingdon, UK) at 4 °C overnight, then in horseradish peroxidase (HRP)-labelled anti-mouse Ig (1:1000) (Dako; Glostrup, Denmark) for 1 h at RT and finally in Luminata Forte Western HRP Substrate (Millipore; Massachusetts, USA) for 1 min at RT before imaging (CL-Xposure Film, Thermo Scientific; Illinois, USA). Membranes were washed thrice in PBS-0.05% Tween-20 between incubations.

#### ELISA

Purified human IgA dimer (dIgA) (Nordic-MUbio; Susteren, Netherlands), mouse pIgA (in-house, 3H1-hybridoma [26]), human IgM (Millipore; Billerica, MA), or human IgA serum standard (Nordic-MUbio; Susteren, Netherlands) at 1 μg/ml in pH9 carbonate/bicarbonate buffer diluted four-fold to 0.0625 μg/ml were incubated on 96-well Medisorp Nunc microtiter plates (Thermo Scientific; Waltham, MA) overnight at 4 °C. The cSC (1:5) was added to blocked plates and incubated overnight at 4 °C. Polyclonal sheep anti-human SC (US Biological; Salem, MA) or monoclonal mouse anti-human SC (1 μg/ml) were added to washed plates and incubated for 1 h at 37 °C. HRP-labelled polyclonal donkey anti-sheep (Jackson Immunoresearch; Suffolk, UK) (1:5000) were added and incubated for 30 min at 37 °C. Alternatively, microtiter plates were coated with cSC-CD4 (1:5), incubated overnight at 4 °C and blocked for 1 h at 37 °C to capture aforementioned purified antibodies, followed by HRP-labelled goat anti-human IgA (Abcam; Abingdon, UK) (1:10,000), HRP-labelled anti-human IgM (Millipore; Billerica, MA) (1:5000), or HRP-labelled goat anti-mouse Ig (Dako; Glostrup, Denmark) (1:2000) for 30 min at 37 °C.

Commercial anti-HEV IgM (MP Diagnostics, Singapore) and anti-HCV IgG (Monolisa HCV Plus v2, BioRad; Hercules, California) ELISAs were run according to manufacturer’s protocol and using in-house protocols to detect anti-HCV pIgA, IgA and IgM as described earlier. For anti-HEV and anti-HCV pIgA, sera/plasma were diluted 1:21 or 1:5, respectively, in cSC (1:5) for 1 h, then added to antigen pre-coated wells and incubated overnight at 4 °C. For anti-HCV IgA and IgM detection, plasma samples (1:5) were incubated for 1 h at 37 °C. For anti-HAV pIgA, cSC-CD4 (1:5) or goat anti-human IgM (Jackson Immunoresearch; West Grove, PA) coated microtiter plates were incubated overnight at 4 °C, washed, blocked and sera samples (1:40 for pIgA; 1:80 for IgM) were added to washed plates and incubated overnight at 4 °C. 1 μg/ml HAV pHM-175 antigen (Meridian Life Science; Memphis, TN) was added and incubated for 1 h at 37 °C, then anti-HAV K3-biotin and anti-human IgA1-biotin (1:1000) for 1 h at 37 °C. HRP-labelled streptavidin (Millipore; Billerica, MA) (1:2000) was added and incubated for 1 h at 37 °C.

All assays were run with samples in duplicate, 100 μl/well/incubation and blocked with 200 μl/well/of 1% Bovine Serum Albumin (Sigma-Aldrich; St. Louis, MO)-PBS-0.05% Tween-20, washed thrice with 350 μl/well PBS-0.05% Tween-20, developed with 3,3,5,5′-Tetramethylbenzidine (KPL; Gaithersburg, MD), stopped with 0.5 M H_2_SO_4_ (Sigma-Aldrich; Steinheim, Germany) and read at 450/620 nm.

#### Sera IgM-depletion

To demonstrate that the reactivity observed in cSC assays was not due to cross-reactive IgM, HEV+ sera samples were IgM-depleted using Capture Select™ agarose microbeads following manufacturer’s protocol (Life Technologies; Naarden, The Netherlands).

#### Liver enzymes

Alanine and aspartate aminotransferases (ALT and AST) were measured on Samsung LABGEO Biochemistry Test 15 (Samsung; Gyeonggi-do, Korea) according to manufacturer’s protocol.

#### Statistical analysis

Signal-to-cut-off ratios (S/Co) were calculated using two standard deviations (SD) from mean of uninfected samples. Welch’s t-test was used to analyze cSC binding of different antibodies and Chi-square test for comparison of avidity. Mann–Whitney U test and Pearson correlation analysis were performed to compare antibody reactivity between acute samples and uninfected controls; the Wilcoxon test for non-parametric paired analysis was conducted to determine effect of IgM-depletion on virus-specific pIgA and IgM reactivity in acute samples. Antibody profiles over time were analyzed by Friedman’s non-parametric two-way ANOVA for repeated measures. Analyses were conducted in Microsoft Excel, Stata-11 (StataCorp LP; College Station, TX) and GraphPad Prism-6 (GraphPad Software; La Jolla, CA). p < 0.05 was considered statistically significant.

### Results

#### cSC selectively binds pIgA

**Fig. 1:**
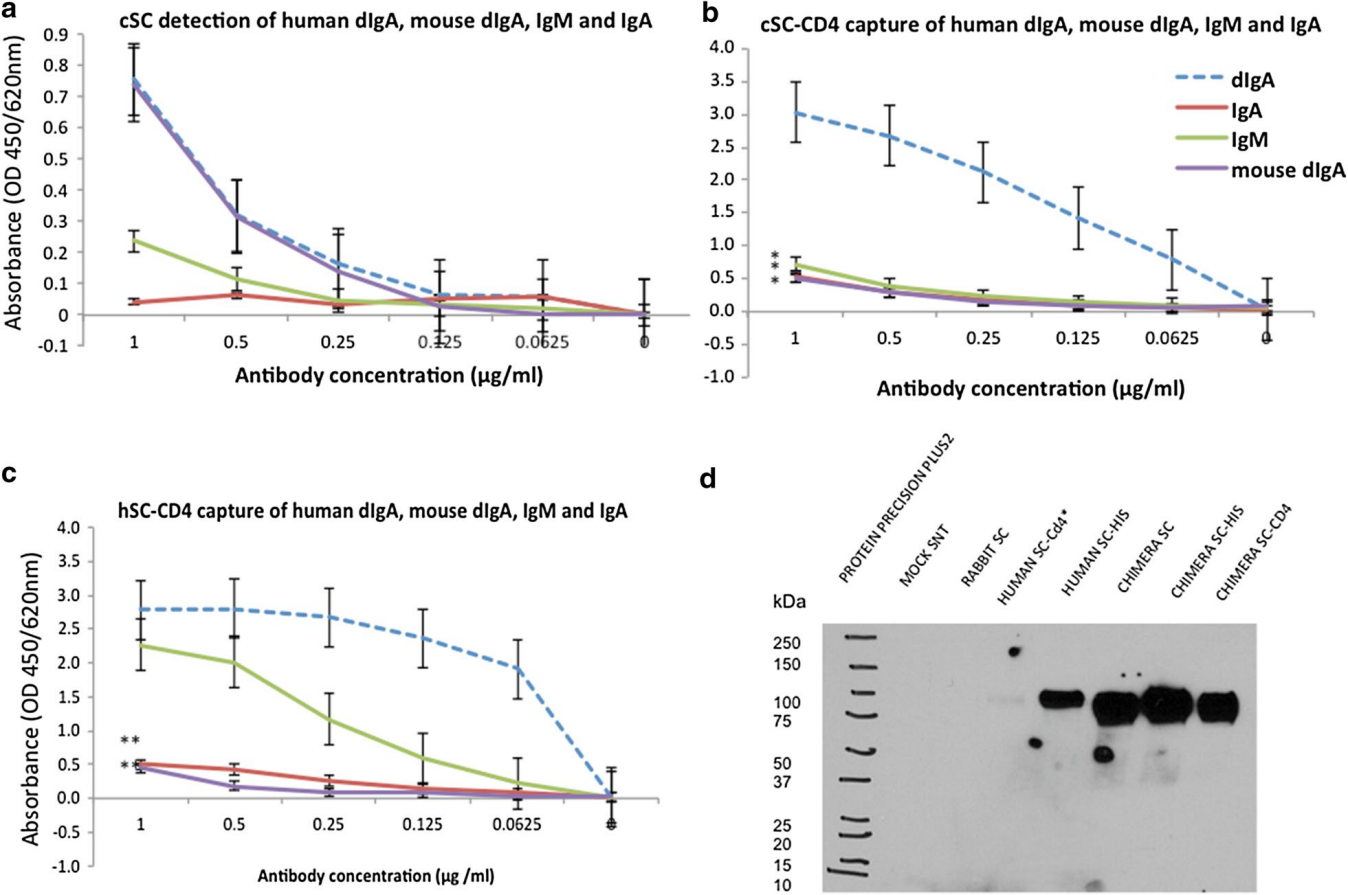
cSC preferential binding to dIgA/pIgA on ELISA and detection with monoclonal anti-human SC. Graphs show **a** cSC detection, **b** cSC-CD4 capture and **c** hSC-CD4 capture, to compare binding and provide dynamic range of cSC and hSC binding to human and mouse dIgA, human IgA and human IgM and **d** monoclonal anti-human SC antibody (AB17377; Abcam, Abingdon UK) detection of 80 kDa hSC and cSC. Note: cSC and hSC not normalized for differences in yield/concentration of active SC, error bars indicate standard error as calculated in Excel. Asterisks indicate statistical significant with reference to dIgA [p value < 0.05 (*), < 0.01 (**), two tailed]

cSC binds > 0.06 μg/ml of purified human and mouse dIgA with negligible cross-reactivity against purified IgM and human IgA, while hSC retains IgM-binding with high reactivity (Fig. 1a–c). Immobilized cSC does not bind mouse dIgA, likely from steric hindrance arising from immobilization. Recombinant SCs are detectable by anti-human SC on immunoblot (Fig. 1d).

#### Anti-HEV and anti-HAV dIgA

**Fig. 2:**
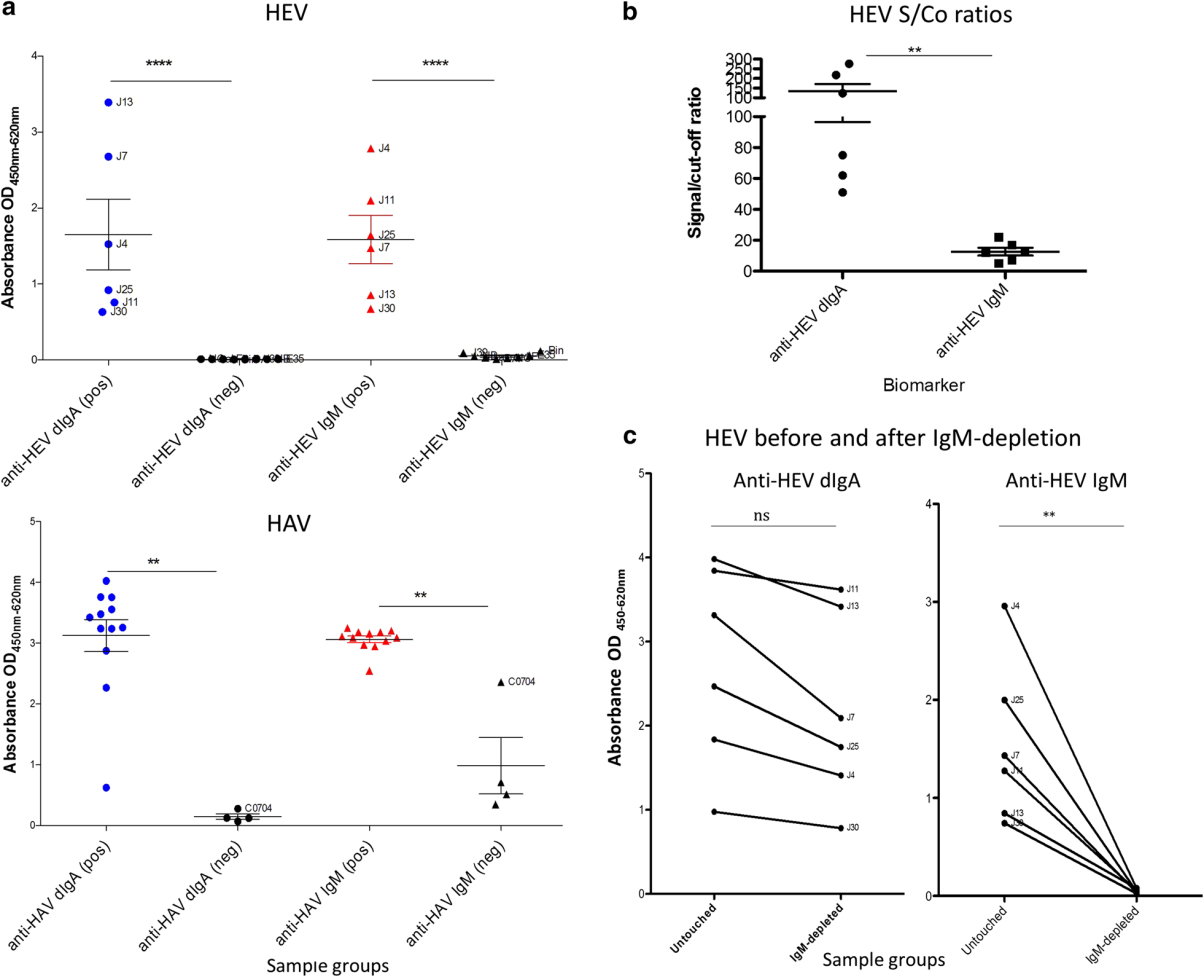
Virus-specific pIgA for serodiagnosis of acute HEV and HAV infection. Scatterplots **a** show anti-HEV and anti-HAV dIgA compared to IgM reactivity detectable in acute and uninfected samples. Graph **b** illustrates the much higher S/Co ratio of dIgA compared to IgM in discriminating acute from uninfected samples, while graph **c** shows anti-HEV dIgA versus anti-HEV IgM in acute samples before and after IgM-depletion, providing evidence that the high reactivity observed for dIgA were not due to cSC cross-reactivity to IgM. Note: Unpaired and paired comparisons conducted using Mann–Whitney U and Wilcoxon test respectively, two-tailed. Asterisks indicate statistical significance of < 0.05 (*), < 0.01 (**), < 0.001 (***), and < 0.0001 (****) and error bars represent SD calculated in GraphPad Prism. S/Co calculated using two SD from mean of uninfected samples

Individuals with acute HEV and HAV infection have significantly higher levels of anti-HEV and anti-HAV pIgA, respectively, compared to uninfected controls (HEV: p < 0.001; HAV: p = 0.001) (Fig. 2a). Levels of anti-HEV and anti-HAV pIgA were comparable to anti-HEV and anti-HAV IgM, but with higher background reactivity from uninfected samples observed for IgM. In particular for HAV, an uninfected control immunized with intramuscular HAV vaccine 2 weeks prior (C0704), exhibits high reactivity for anti-HAV IgM but negligible reactivity for anti-HAV pIgA. The low correlation between virus-specific pIgA and IgM in acute infection samples for both HEV and HAV (HEV r: − 0.25, 95% CI − 0.88 to 0.71, p = 0.636; HAV r: 0.05, 95% CI − 0.54 to 0.60, p: 0.885) suggests that pIgA production is independent of IgM in acute phase response, and may have higher diagnostic potential based on higher S/Co observed (Fig. 2b). While IgM-depleted samples have slight reduction of anti-HEV pIgA (paired test p = 0.016; unpaired test p = 0.394), anti-HEV IgM is undetectable after IgM-depletion (paired test p = 0.007; unpaired test p = 0.002) (Fig. 2c), supporting the pIgA-specific nature of the cSC.

#### Anti-HCV pIgA serological profile over time

**Fig. 3:**
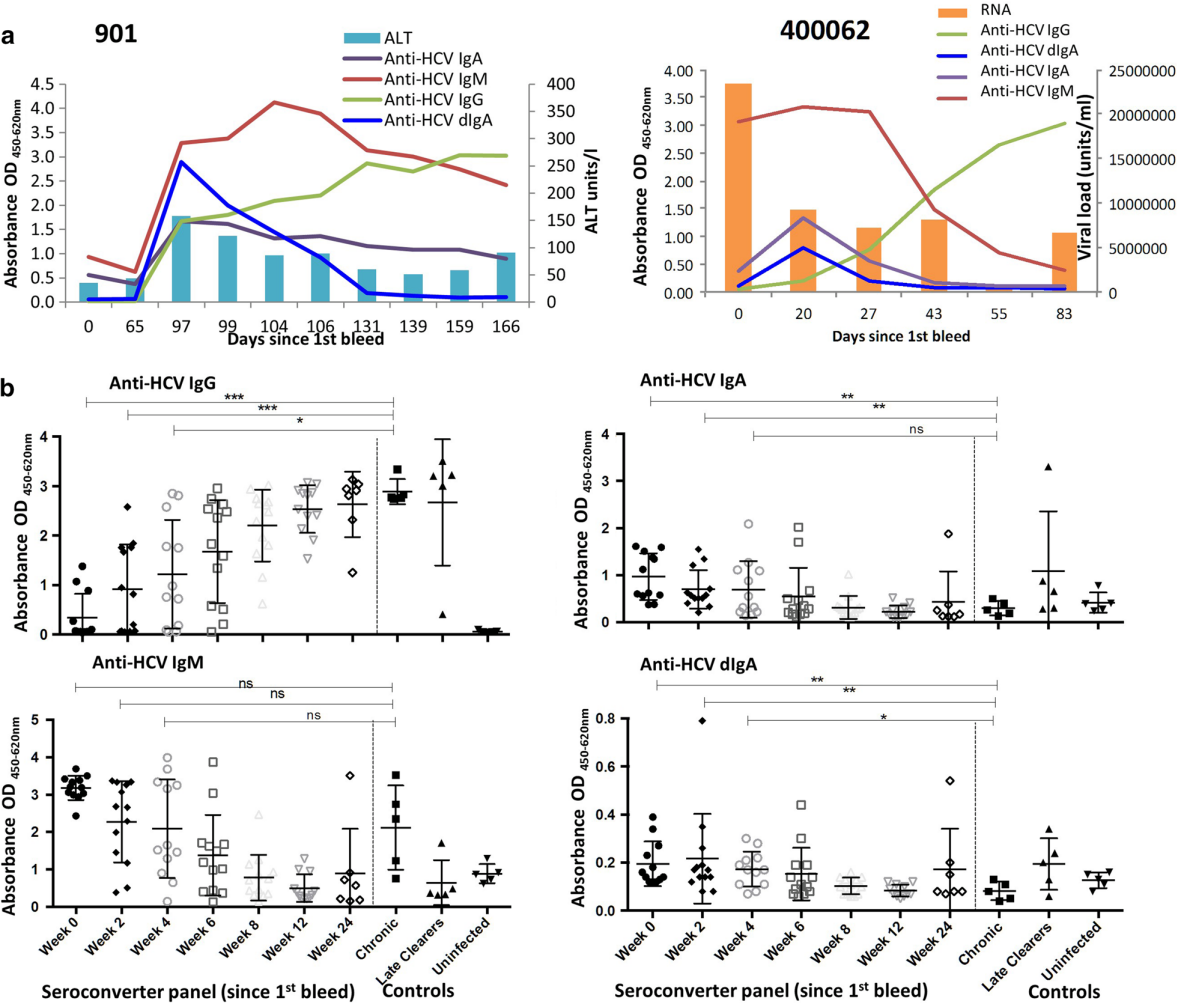
HCV serological profile over time. **a** Graphs of anti-HCV antibodies compared to viral RNA or ALT over time in two seroconversion panels demonstrate that dIgA may have a different profile compared to IgA (panel 901) and it disappears even in ongoing infection (panel 400062). **b** Scatterplots of anti-HCV reactivity in seroconversion panels show that unlike anti-HCV IgG and IgM, anti-HCV pIgA (and to some degree anti-HCV IgA) to be statistically significantly higher in early acute phase (0–4 weeks since 1st bleed) compared to chronically infected patients. Note: Unpaired comparisons between each acute phase/early incident timepoint and chronic samples conducted using Mann–Whitney U, two tailed with Bonferonni adjustment in GraphPad Prism

Anti-HCV IgG increased over time and was higher in later timepoints and in patients who were chronically infected or cleared the infection after 6 months. Anti-HCV pIgA declined over time even in ongoing infections—unlike anti-HCV IgA and ALT, which may persist, as observed in panel 901 and 400062 (Fig. 3a). Anti-HCV pIgA and IgA in acutely infected individuals were significantly higher at week-0 (p: 0.003), week-2 (p: 0.006) and week-4 (p: 0.022) post-1st bleed compared to chronically infected individuals. In contrast, anti-HCV IgG is significantly lower in acutely infected individuals at week-0 (p < 0.001), week-2 (p < 0.001), and week-4 (p: 0.014) compared to chronically infected individuals (Fig. 3b). These observations suggest that pIgA is produced predominantly during the acute phase even for infections that may progress to chronicity.

### Discussion

Previous detection of antigen-specific pIgA in serum of individuals infected with rubella, measles and varicella required physical separation of pIgA from IgA [19], and did not assess diagnostic potential. This study highlights use of recombinant cSC for measuring serum pIgA by ELISA (including in zoonotic hosts as binding is conserved among tetrapods [27]), and virus-specific pIgA as a novel biomarker of acute hepatotrophic infections. Data from patients with acute HAV, HCV and HEV infection suggests: (1) virus-specific pIgA is detectable in patients during the acute phase of infection (Fig. 2); (2) transient profile of serum anti-HCV pIgA may differ from total anti-HCV IgA, and discriminate acute from chronic HCV infections (Fig. 3B); (3) anti-HEV pIgA is not correlated with and has higher specificity than anti-HEV IgM; and (4) anti-HAV pIgA is undetectable in HAV-vaccinated individual and may be more specific to natural infection.

Anti-HEV IgA is reportedly a potential marker of acute HEV infection [28–30], although it persists > 30 days longer than IgM [29]. In contrast, pIgA is known to have a shorter half-life in serum [31], which may serve as a better marker of recent infection. Unlike anti-HCV IgM, detectable in 51–82% of patients after 6 months and beyond [32, 33], data from HCV seroconversion panels suggest that pIgA detected in acute phase is transient, even in an ongoing infection. Low correlation between virus-specific IgM and pIgA and varying propensity of antibody-isotype response observed may relate to the duration of infection, and/or to the proportion of antigens circulating systemically or localized to the liver [34]. Detection of virus-specific pIgA may complement existing ELISA and rapid immunochromatographic assays for acute viral hepatitis infections [35, 36], which merits further investigation.

## Limitations

Although highly dynamic, the source of serum virus-specific pIgA, either from mucosal production [37, 38] or produced in sudden response to antigens [31, 39–41], remains contentious. Serodiagnostic measure of pIgA used limited numbers of samples and controls, and without a standard curve. Application in other diseases of interest may depend on the route of transmission and the major site(s) of pathogen replication, with limited diagnostic use in patients with IgA nephropathy glomerulonephritis due to increased production of antigen-specific pIgA [42, 43]. The signal of anti-HCV pIgA in these samples were low compared to anti-HEV and anti-HAV pIgA, but may be amplified through biotinylation.

## Abbreviations

ALT: alanine transferase
AST: aspartate aminotransferases
cSC: chimeric secretory component
D: domain
dIgA: dimeric immunoglobulin A
DMEM: Dulbecco’s Modified Eagle Medium
ELISA: enzyme-linked immunoassay
HAV: hepatitis A virus
HCV: hepatitis C virus
HEK: human embryonic kidney
HEV: hepatitis E virus
HRP: horseradish peroxidase
hSC: human secretory component
Ig: immunoglobulin
PBS: phosphate buffered saline
pIgA: polymeric immunoglobulin A
pIgR: polymeric immunoglobulin receptor
RNA: ribonucleic acid
rSC: rabbit secretory component
S/Co: signal-to-cut-off
SC: secretory component
SD: standard deviation
SIgA: secretory immunoglobulin A
